# European Public Health News

**DOI:** 10.1093/eurpub/ckad159

**Published:** 2023-10-10

**Authors:** Marieke Verschuuren, Nino Berdzuli, Ketevan Kandelaki, Marcello Gelormini, Tine Rikke Jorgensen, Danilo Lo Fo Wong, Regien Biesma Blanco, Floris Barnhoorn, Marieke Verschuuren

**Affiliations:** EUPHA; Director Country Health Programmes WHO Regional Office for Europe; Technical Officer AMR WHO Regional Office for Europe; Technical Officer AMR WHO Regional Office for Europe; Technical Officer AMR WHO Regional Office for Europe; Regional Adviser Control of AMR WHO Regional Office for Europe; EPH Conference; EPH Conference; EPH Conference

In this edition of the European Public Health News, Verschuuren says goodbye as Executive Director at EUPHA, while looking back at EUPHA’s achievements over the past year. Berdzuli *et al*. from WHO Regional Office for Europe address the issue of antimicrobial resistance (AMR) and look ahead to the new regional roadmap on AMR for 2023–30, which will be up for adoption at the 73rd Regional Committee session in Kazakhstan this year, and aims to bridge the gap between knowledge and action on AMR. Finally, Biesma Blanco *et al*. present the Dublin statement, related to the forthcoming European Public Health Conference in November. In this statement, the European Public Health conference, EUPHA and national public health associations and organizations across Europe, call upon European leaders to take transformative action towards a more environmentally friendly, resilient and healthy future.

## EUPHA: what a small secretariat can achieve when it can build on a large, dedicated expert network

VerschuurenMarieke
EUPHA

It is early August when I am writing this column, and at the office, in between staff members being away for summer holidays, we are working on an activity report for the European Commission for the operating grant that EUPHA received for the past 12 months. Although sometimes tedious, I also find this kind of work rewarding, as it provides insight into everything that has been achieved. In a busy organization like EUPHA, there often is not much time for reflection, but it is also good to dwell on accomplishments once in a while! I have to say that I find it pretty amazing how much EUPHA is able to do with its small secretariat, together with its vast network of experts across Europe.

Just to highlight a few eye-catching examples: during the operating grant period, EUPHA representatives participated in over 40 meetings, events and conferences; EUPHA activities, supported by our sections, at the European Public Health Conference 2022 in Berlin included (co-)organization of one plenary, 11 pre-conferences, 27 Join The Network meetings and 70 workshops; EUPHA published or supported more than 20 position papers, statements and letters; more than 100 students and early career professionals applied for the new EUPHAnxt mentoring programme; EUPHA initiated and coordinated the European Public Health Week (EUPHW) 2023 with over 220 events in 39 countries; the Impact Factor of this Journal increased from 3.367 to 4.424; and due to increased social media activity, in particular during our dedicated campaigns for the EUPHW and EUPHA’s activities at the conference, the number of followers on our social media channels have been steadily increasing, to almost 8700 on Twitter and about 8450 on LinkedIn currently.

This column marks my last column as the EUPHA Executive Director. I have had this role for a year now, and I have come to the conclusion that I am not the best fit for this function, and vice versa. Nevertheless, I have very much enjoyed and appreciated working with all the EUPHA colleagues, both the office staff and all the active experts in the EUPHA network. It is really encouraging and exciting to see what people can achieve when they join forces for a common cause, especially taking into account that it often requires time on top of a day job and outside of office hours; the voluntary efforts made by all the EUPHA experts are really commendable.

Of course, there is still room for improvement and things on the wish list. Nevertheless, I am leaving a vibrant organization, that no doubt will continue to thrive and grow under the management of my successor, together with all the members of the EUPHA family. I thank everyone for the pleasant collaboration, and I am sure I will cross paths with many of you again somewhere in Europe in our quest to improve population health!

## Antimicrobial resistance in the WHO European Region—bridging the gap between knowledge and action

BerdzuliNino
Director Country Health Programmes WHO Regional Office for Europe
KandelakiKetevan
Technical Officer AMR WHO Regional Office for Europe
GelorminiMarcello
Technical Officer AMR WHO Regional Office for Europe
JorgensenTine Rikke
Technical Officer AMR WHO Regional Office for Europe
Lo Fo WongDanilo
Regional Adviser Control of AMR WHO Regional Office for Europe

The thought of an era of untreatable infections; a complex, grave international health security threat, should propel us to act with force. Resistant infections have pandemic potential. But it hasn’t yet. Not to the extent needed.

Since the microbiologist Alexander Fleming discovered penicillin in 1928, antibiotics have revolutionized medical science and saved countless lives. But Fleming also cautioned that misuse would result in microbes becoming resistant to treatment; in his own words, an evil he hoped could be averted. Nearly 100 years on, antimicrobial resistance (AMR) is one of the top 10 global public health threats facing humanity. It threatens economies, international trade, food systems, the very functionality and resilience of our health systems. It compromises cornerstones of universal health coverage; patient safety and access to quality service across the care continuum.

The facts speak for themselves. According to the GRAM Project’s estimates, in 2019, bacterial AMR in the WHO European Region was associated with over half a million deaths.^1^ The OECD has also pointed out that the health burden of infections due to bacteria with AMR in the EU/EEA population, is comparable to that of influenza, tuberculosis and HIV/AIDS combined.^2^ As a region, we have gained a lot of experience. The effectiveness of certain interventions has been mounting. The WHO European strategic action plan, supported by WHO and partners, prioritized AMR in countries outside the EU. A number of countries were at the helm of putting AMR firmly on the agenda of the WHO European Region and the European Union, both of which launched action plans in 2011. Since then, several EU Presidencies—among them the Trio Presidency of the Council of the EU, consisting of France, Czech Republic and Sweden—have put special emphasis on AMR. France advocated for a One Health approach, whereas Sweden focused on ensuring sustainable access to existing antibiotics and stimulating the development of new ones. The current presidency; Spain, Belgium and Hungary, is now entrusted with maintaining that focus. As well as the following presidency, and the one after that.

Today, nearly all European countries have an AMR action plan. The downside is slow and fragmented implementation of evidence-based interventions, conflicting priorities and the fact that more than 74% of these action plans does not have an allocated budget. In other words, we have progressed, but not enough. Action has been incomprehensive and too slow. At the root of that, posing a grave threat to the nearly 1 billion people living in our Region, is an alarming disconnect between what we know about AMR and what we are doing to act on it. In an era characterized by multiple overlapping and interconnected health threats; an era of global boiling as the UN Secretary General framed it in July this year, that disconnect needs to be addressed. National AMR action plans should be linked to national security plans. And interventions that have proven to work need to be implemented, fast.

This coming October, Member States of the WHO European Region will convene at our 73rd Regional Committee session in Kazakhstan, where they are invited to adopt a new regional roadmap on antimicrobial resistance for 2023–30, a blueprint aligned to country specificities, linked to sub-regional, European and global strategies. The goal? To inspire and support WHO member states to identify and implement evidence-based, sustainable interventions—with targets tailored to local contexts. In short, to bridge the gap between knowledge and action on AMR.

The roadmap provides tools, proposes people-centred interventions, and aims to reduce the AMR burden through a holistic lens comprising of e.g. gender and inequities. It positions AMR in a broader context of health system resilience and emergency preparedness efforts. It accelerates implementation as well as strengthening the systems that can deliver solutions. It involves multiple sectors through a One Health approach, because antimicrobials are not only used to treat and prevent diseases in humans but also in animals and crops. It lays out action areas with evidence-based interventions on prevention and control, environment and social determinants, stewardship, community awareness and enabling behaviours. It includes tools and pathways that enable the implementation of these action areas; that is regulations, legislations, governance, leadership, surveillance, laboratories, workforce, innovation, digital technology and research—and far more robust and sustained than we have seen until now.

The AMR Compass is one of the roadmap’s highlights, serving as a diagnostic tool, allowing stakeholders to assess their country’s needs, strengths and readiness and facilitate policy dialogue and national target-setting, in line with the Sustainable Development Goals. The Novel Medicines Platform is also part of the roadmap, because access to medicines; to existing antimicrobials, is a vital component on the road ahead.

Operationalizing and sustaining progress on the roadmap, can only be done through broad, inclusive dialogue and partnerships, such as the ones forged at the upcoming WHO Europe Regional Committee Meeting in October. And because AMR transcends borders, sectors, ethnicities and political divides, the success of the roadmap depends on the active engagement of governments, partners, the private sector, non-governmental organizations, civil society, academia, investment banks. All stakeholders. All of us. It is high time.

## Our Food, Our Health, Our Earth: A Sustainable Future for Humanity

Biesma BlancoRegien
EPH Conference
BarnhoornFloris
EPH Conference
VerschuurenMarieke
EPH Conference

From 8 to 11 November, we will meet in Dublin, Ireland, for the 16th European Public Health Conference. This year’s conference takes place at a critical turning point in history, where the interconnection between food systems, health and the earth requires urgent and sustainable action to protect our planet and all its inhabitants. As at every EPH conference, we are launching a conference statement calling on European leaders to take action to pave a sustainable path forward and secure a healthier, more resilient future for future generations. We invite (public) health organizations and associations to co-sign and support the declaration. The final version of the statement is available on the websites of EUPHA and EPH Conference.

### Outline for the Dublin 2023 statement

The consequences of our current food system are far-reaching, affecting both human health and the environment. From deforestation and greenhouse gas emissions to water pollution and biodiversity loss, our choices in food production, distribution and consumption directly affect the Earth's health. Equally concerning is the impact on human well-being, with rising rates of diet-related diseases and malnutrition threatening the lives of millions.

We can no longer overlook the undeniable link between these challenges. Embracing a holistic approach that prioritizes sustainability within our food system is crucial for fostering human health but also the health of ecosystems and the overall environment. Now is the time for individuals, communities, businesses and governments to unite and implement sustainable practices across the entire food supply chain. By supporting regenerative agriculture, reducing food waste, promoting plant-based diets and emphasizing local and seasonal produce, we can pave the way towards a more environmentally friendly and resilient future.

The Budapest Declaration, adopted during the 7th Ministerial Conference on Environment and Health in Hungary in 2023, serves as a powerful wake-up call for urgent action in addressing health challenges linked to climate change, pollution, biodiversity loss and land degradation. Especially in the aftermath of the COVID-19 pandemic, it is imperative that we respond to this call and take decisive steps to safeguard our planet and well-being. The European Green Deal is a comprehensive and ambitious policy initiative launched by the European Commission in December 2019. It offers a transformative vision for the EU, aiming to transition into a sustainable, climate-neutral and environmentally responsible economy by 2050. To achieve these objectives, the European Green Deal includes various legislative and regulatory measures, financial instruments and initiatives designed to combat climate change, protect the environment and create a more sustainable future for all EU citizens. A critical component of the European Green Deal is the Farm to Fork Strategy, aimed at establishing a fair, healthy and environmentally friendly food system. Embracing sustainable practices, combating greenhouse gas emissions, reversing biodiversity loss and prioritizing food security and nutrition for all are crucial steps towards building a more sustainable food system.

To realize this transformative vision, the European Public Health conference, EUPHA and national public health associations and organizations across Europe, call upon European leaders to take the following actions:

- Embrace a holistic approach to health and systems thinking, prioritizing sustainable solutions that acknowledge the interplay between our food, health and the health of our planet. By recognizing this interconnectedness we can make informed and responsible choices.
- Enact and enforce legislation that promotes sustainable agriculture, ensures food security for all and safeguards our natural ecosystems. It is essential for businesses to accept their environmental responsibility, prioritizing sustainable sourcing and operations. As individuals, we must make conscious choices in our daily lives, from the foods we consume to the products we purchase.
- Education and awareness play an instrumental role in empowering individuals to make informed decisions and advocate for change. By promoting the vital link between the food system, health and planetary health, we can inspire a collective commitment to a sustainable future for humanity.
- Accelerate intersectoral action, breaking down silos, and embracing visionary leadership and innovative governance models to make meaningful progress. Promote collaboration and integration across various sectors including agriculture, health, environment and education. By working together we can bring together diverse perspectives, expertise and resources to develop comprehensive strategies and create synergies to collectively tackle complex challenges.

Now is the time for action. Together, with urgency and determination, let us work towards building a food system that nurtures both our bodies and our planet. United, we can forge a sustainable path forward, securing a healthier, more resilient future for generations to come. The moment to act is now, and we must seize this opportunity to safeguard the well-being of our world and all of humanity.

We look forward to seeing you all in person, in Dublin.
